# Data on endogenous bovine ovarian follicular cells peptides and small proteins obtained through Top-down High Resolution Mass Spectrometry

**DOI:** 10.1016/j.dib.2017.05.042

**Published:** 2017-05-26

**Authors:** Valérie Labas, Ana-Paula Teixeira-Gomes, Laura Bouguereau, Audrey Gargaros, Lucie Spina, Aurélie Marestaing, Svetlana Uzbekova

**Affiliations:** a UMR PRC, INRA 85, CNRS, Université de Tours, IFCE, 37380 Nouzilly, France; b UMR ISP, INRA, Université de Tours, 37380 Nouzilly, France; c INRA, Plateforme d’Analyse Intégrative des Biomolécules, Laboratoire de Spectrométrie de Masse, 37380 Nouzilly, France; d INSA/CNRS 5504 - UMR INSA/INRA 792, Toulouse, France

**Keywords:** Top-down proteomics, Bovine, Ovary, Follicular cells

## Abstract

The endogenous peptides and small proteins extracted from bovine ovarian follicular cells (oocytes, cumulus and granulosa cells) were identified by Top-down High Resolution Mass Spectrometry (TD-HR-MS/MS) in order to annotate peptido- and proteoforms detected using qualitative and quantitative profiling method based on ICM-MS (Intact Cell Matrix-Assisted Laser Desorption/Ionization Time-of-Flight Mass Spectrometry). The description and analysis of these Top-down MS data in the context of oocyte quality biomarkers research are available in the original research article of Labas et al. (2017) http://dx.doi.org/10.1016/j.jprot.2017.03.027[1]. Raw data derived from this peptidomic/proteomic analysis have been deposited to the ProteomeXchange Consortium via the PRIDE partner repository (dataset identifier PXD004892). Here, we described the inventory of all identified peptido- and proteoforms including their biochemical and structural features, and functional annotation of correspondent proteins. This peptide/protein inventory revealed that TD-HR-MS/MS was appropriate method for both global and targeted proteomic analysis of ovarian tissues, and it can be further employed as a reference for other studies on follicular cells including single oocytes.

**Specifications Table**

**Table:** 

Subject area	Oocyte maturation
More specific subject area	Bovine (Bos Taurus) follicular cells peptide/protein repository
Type of data	Raw and processed/analyzed mass spectrometry data obtained by Top-down high resolution mass spectrometry
How data was acquired	High Resolution Mass Spectrometry (HR-MS/MS) analyses of protein extracts performed by : 1) direct infusion to a LTQ orbitrap Velos Mass Spectrometer (ThermoFisher), 2) µLiquid Chromatography (µLC) using an Ultimate®3000 Ultra High Pressure Liquid Chromatographer combined to HR-MS/MS, 3) µLC-HR-MS/MS with pre-fractionations based on Reverse Phase-High Pressure Liquid Chromatography (RP-HPLC) or gel filtration separation methods.
Data format	Raw data
Experimental factors	Processed and analyzed data using ProSight PC : ProSight Upload Format (PUF)
Experimental features	Immature and mature bovine follicular cells
Data source location	High throughput identification of endogenous peptides and small proteins from oocytes, cumulus and granulosa cells
Data accessibility	http://www.ebi.ac.uk/pride/archive/projects/PXD004892
	ftp://ftp.pride.ebi.ac.uk/pride/data/archive/2017/04/PXD004892

**Value of the data**

• The data presents the first inventory of endogenous peptides and small proteins identified in bovine ovarian follicular cells (oocytes, cumulus and granulosa cells) to annotate biomolecules detected by ICM-MS on whole follicular cells including single oocytes. The data could be used by others researchers in reproduction sciences for biomarker research.
• The data obtained using analysis based on three Top-Down HR-MS/MS approaches (direct infusion, µLC-HR-MS/MS with or without pre-fractionations) allows other researchers to choice a strategy adapted to their biologic models.
• The identifications of the proteoforms and peptidoforms from granulosa cells protein extract by combining µLC-HR-MS/MS and four different pre-fractionation strategies (3 separation methods based on reverse phase chromatography and one on gel filtration chromatography) could be compared to others separation and MS identification methods.

## 1 Data

**Fig. 1:**
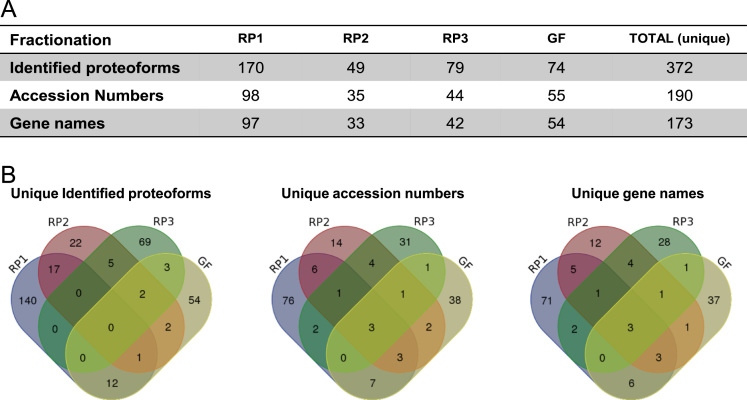
Distribution of the peptido- and proteoforms, accession numbers and gene names identified for the RP and GF fractionations. (A) The number of biomolecules, UniprotKB accession numbers and genes identified by top-down high-resolution MS for each fractionation (RP1, RP2, RP3 or GF), resulting in 372, 190, 173 unique values, respectively. (B) Venn diagram showing the distribution of unique peptido- and proteoforms, accession numbers and gene names identified for the four separation methods.

This dataset represents a list of peptidoforms and proteoforms extracted from bovine ovarian follicular cells and identified by TD HR-MS/MS. They correspond to molecular species previously characterized by ICM-MS on whole follicular cells [1]. Depend upon the available quantity of biological materials biomolecules were analyzed by HR-MS/MS using three approaches:

1) Direct infusion of the proteins prepared from oocyte-cumulus complexes (OCCs); 15 biomolecules were identified (Supplementary data Table DB1-A).
2) Using µLC-HR-MS/MS, direct injection of protein extracts obtained from OCCs, oocytes and cumulus cells (CC) at different stages and granulosa cells (GC); 52 biomolecules were identified (Supplementary data Table DB1-B).
3) Using µLC-HR-MS/MS with pre-fractionations (3 separation methods based on reverse phase (RP) and 1 on gel filtration (GF) chromatography) of proteins from large pool of GC; 372 biomolecules were identified (Supplementary data Table DB1-C). For RP1, RP2, RP3 and GF separation methods, 170, 49, 79 and 74 non-redundant biomolecules corresponding to 98, 35, 44 and 55 UniprotKB accession numbers and 97, 33, 42 and 54 gene names, respectively, were identified (Fig. 1A). Comparison between the four separation methods is shown (Fig. 1B).

**Fig. 2:**
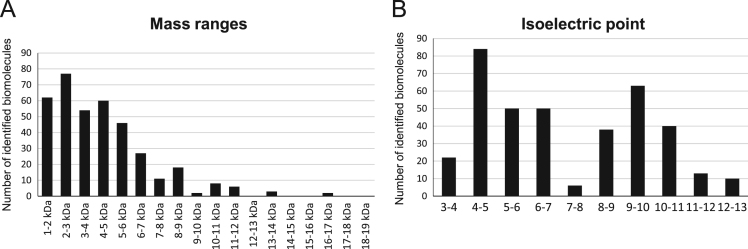
Distribution of the 386 peptido- and proteoforms identified in follicular cells by the top-down proteomic approach, including the mass range and the isoelectric point. (A) The representation of the number of identified biomolecules within the 1000–17,000 mass range showing a bias, as biomolecules <10 kDa were easier to identify with these conditions. (B) The distribution of isoelectric points (pI) showing an equivalent number of very acidic and very alkaline biomolecules.

**Fig. 3:**
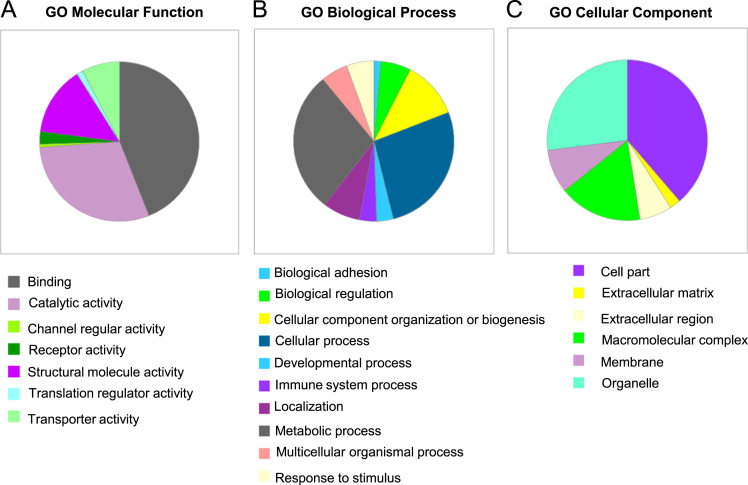
Molecular function (A), biological process (B) and cellular compartments (C) of the biomolecules identified in bovine follicular cells by Top-Down High Resolution Mass Spectrometry, according to the Gene Ontology classification.

In total, 386 different intact proteins or fragments corresponding to 194 genes were identified (Supplementary data Table DB1-D). The distribution of molecular weight and isoelectric point of the identified masses is represented in Fig. 2A and B, respectively. Functional annotation of identified proteins was performed using Panther Functional Classification System (http://www.pantherdb.org/), GeneAnalytics and Database for Annotation, Visualization and Integrated Discovery (https://david.ncifcrf.gov/) (Fig. 3 and Supplementary data Table DB2).

## 2 Experimental design, materials and methods

This dataset was produced with the objective to identify, using TD HR-MS/MS, the peptides and small proteins which have been previously characterized by ICM-MS on whole bovine ovarian follicular cells and especially on intact single oocytes. Due to partial similarity of protein ICM-MS profiles between the oocytes, cumulus cells (CC) and granulosa cells (GC) (39.5% oocyte peaks are common with CC and 45.5% with GC), it seemed pertinent to develop TD HR-MS/MS approach using these three cellular types as source. Depending on the amount of available biological material (abundant or not), we have therefore performed three different TD HR-MS/MS approaches a) direct infusion, b) injection on µLC-HR-MS/MS system of total protein extracts, c) combine µLC-HR-MS/MS with four off-line pre-fractionations of protein extract from GC. Thus, in order to reduce sample heterogeneity and increase the number of identified proteins, we have combined reverse phase (RP) or gel filtration liquid chromatography separation methods with µLC-HR-MS/MS. Top-Down High Resolution Mass Spectrometry detailed protocols and identification parameters for each approach are provided in supplementary data DB3.

## Appendix A Supplementary material

Supplementary material: **Table DB1** : List of top-down HR-MS/MS identifications for direct infusion (A), direct injection of protein extracts to µLC-HR-MS/MS (B), µLC-HR-MS/MS after RP1, RP2, RP3 and GF pre-fractionations (C), and for all combined strategies (D)..

.

Supplementary material: **Table DB2.** Gene Ontology analysis of the proteins identified by top-down HR-MS/MS in bovine ovarian follicular cells..

.

Supplementary material: **DB3.** Top-Down High Resolution Mass Spectrometry detailed protocols and identification parameters for a) direct infusion, b) µLC-HR-MS/MS without pre-fractionation, c) µLC-HR-MS/MS with pre-fractionations.
