# Supplementary material for: Data on endogenous bovine ovarian follicular cells peptides and small proteins obtained through Top-down High Resolution Mass Spectrometry
Source: Data Brief. 2017 May 26;13:175–9. doi: 10.1016/j.dib.2017.05.042 (PMC5454127; doi:10.1016/j.dib.2017.05.042)
Supplement: Supplementary file 1 — Supplementary material [file mmc1.docx]

Authors declared no conflicts of interest.
