# Supplementary material for: Data on endogenous bovine ovarian follicular cells peptides and small proteins obtained through Top-down High Resolution Mass Spectrometry
Source: Data Brief. 2017 May 26;13:175–9. doi: 10.1016/j.dib.2017.05.042 (PMC5454127; doi:10.1016/j.dib.2017.05.042)
Supplement: Supplementary file 4 — Supplementary material: DB3. Top-Down High Resolution Mass Spectrometry detailed protocols and identification parameters for a) direct infusion, b) µLC-HR-MS/MS without pre-fractionation, c) µLC-HR-MS/MS with pre-fractionations. [file mmc4.docx]

**Supplementary data DB3 : Top-Down High Resolution Mass Spectrometry detailed protocols and identification parameters**

1. TD HR-MS/MS by direct infusion

Forty immature bovine oocytes-cumulus complexes (OCCs) were collected and rinsed with Phosphate-buffered saline (PBS) and Tris-Sucrose buffer (TSB : 20 mM Tris-HCl, pH 6.8, and 260 mM sucrose). After frozen-thaw process, peptides and proteins (about 40 µg) were extracted using Tris-Urea (TU) buffer (6 M Urea, 50 mM Tris-HCl pH 8.8 buffer containing protease inhibitor cocktail (Sigma-Aldrich, Saint-Quentin Fallavier, France)). After centrifugation, supernatant was acidified using formic acid 5%. Molecular species were desalted and concentrated using ZipTip C4 solid-phase extraction (ZT-C4-SPE) (Millipore Corporation, Billerica, MA) with formic acid (FA) 1% and enriched by a differential elution with acetonitrile (ACN). Fractions eluted with 20% ACN/80% H2O in presence of formic acid (FA) 1% were dried using speedVac 1010 (Thermofisher). Micropurified species were dissolved with 5µL of 50% MetOH/50% H2O in presence of FA 1% and loaded into a metalized nanoelectrospray needle (PicoTip emitters, New Objective).

All experiments were performed using a dual linear ion trap Fourier Transform Mass Spectrometer (FT-MS), on a LTQ Orbitrap Velos instrument (Thermo Fisher Scientific, Germany) operating in positive mode. Data were acquired using Xcalibur software v2.1 (Thermo Fisher Scientific, San Jose, CA). Standard mass spectrometric conditions for all analyses were spray voltage 1.1-1.4 kV, no sheath and auxiliary gas flow; heated capillary temperature, 275 °C; predictive automatic gain control enabled, and an S-lens RF level of 60%. Source fragmentation energy was set at 10 V. All analyses were performed manually both the FT-MS spectrum using the profile mode in the 400-2000 m/z mass range and the MS/MS spectrum using HCD (High energy Collisional Dissociation) and/or CID (Collision Induced Dissociation). Target resolution was 100,000 for MS and MS/MS analysis. The selected precursor width for fragmentation was 2-3 m/z. Spectra corresponded to the accumulation of scans over approximately 1 min, yet good signal to noise ratios could be obtained within less time.

Identification and structural characterization were performed using ProSight PC software 2.0 (Thermo Scientific, San Jose). Raw data files were individually processed by THRASH algorithm (signal/noise: 2-3) to convert each fragmentation multi-charged scan into monoisotopic neutral mass values. Individual ProSight PC experiment was created for each biomolecules generating an XML file in ProSight Upload Format (PUF). From PUF files, searches were performed using the “Absolute Mass and Biomarker” search options against a database made via shotgun annotation from the UniprotKB Swiss-Prot Bos taurus release 2015_06 (bos_taurus_2015_06_top_down_simple.pwf) downloaded from ftp://prosightpc.northwestern.edu/). Searches were performed using the following parameters: 5 ppm mass tolerance for the monoisotopic precursors, 5 Da for the average precursor, 15 ppm for fragment ions mass tolerance, the delta mass feature deactivated, with activated mode for disulfide bridges and N-terminal post-translation modifications (acetylation and initial methionine cleavage). Proposed sequences with E-value < 1 x 10^-6^ were considered positively identified with a minimum of 10 matching fragment ions. Interpretation was performed with a manual, iterative process with variable parameters independently tested to maximize the E-value.

1. µLC-HR-MS/MS without pre-fractionation

Total proteins were extracted from pools of granulosa cells (GC), OCCs (n = 300), cumulus cells (CC) and oocytes both at immature (n = 200) and mature (n = 140) state using TU buffer as earlier described. Protein concentration was determined using the DC Protein Assay (Bio-Rad, Marnes-La-Coquette, France). Around 10-15 µg of protein total extract were desalted and concentrated using ZT-C4-SPE. All samples were reconstituted in 10 μL of formic acid 1%, sonicated, and then analyzed by on-line micro-liquid chromatography tandem mass spectrometry (µLC-MS/MS) on the LTQ Orbitrap Velos mass spectrometer (Thermo Fisher Scientific, Germany) coupled to an Ultimate® 3000 RSLC Ultra High Pressure Liquid Chromatographer (Dionex, The Netherlands) controlled by Chromeleon Software (version 6.8 SR11; Dionex, The Netherlands). Eight microliters of each sample were injected using µL-pickup mode and loaded on a Dionex trap column (Monolithic PS-DVB PepSwift, 200 µm inner diameter x 5 mm long). Solution A was composed of 2% acetonitrile in water in presence of 0.1% formic acid, whereas solution B consisted of 16% water in acetonitrile and 0.1% formic acid. Biomolecules were pre-concentrated for 10 min at 10 µL/min with 4% solvent B. The separation was conducted using a Dionex column (Monolithic PS-DVB PepSwift, 200 µm inner diameter x 5 cm long). The flow rate was set to 1 µL/min. The gradient consisted of 4-10% B for 1 min, 10-95% B for 60 min, 75 to 99% B for 1 min, constant 99% B 20 min and return to 4 % B in 1 min. The column was re-equilibrated for 15 min at 4% B between runs. The eluate was sprayed using a SilicaTip emitter with 30 μm inner diameter and 360 µm outer diameter (New Objective, Woburn, MA, USA) into a Thermo Finnigan Nanospray Ion Source 1.

Standard MS conditions were used as previously described in direct infusion paragraph. Full-MS in profile mode with subsequent data-dependent MS/MS analyses were acquired with a target resolution in the Orbitrap set to 100,000. In the scan range of m/z 400-2,000, the 10 most intense ions were selected and fragmented by HCD (Higher-Energy Collisional Dissociation) with normalized collision energy of 38% and wideband-activation enabled. Ion selection threshold was 500 counts for MS/MS with an isolation width = 3 m/z. The maximum allowed ion accumulation times were 200 ms for full scans (1 microscan) and 200 ms for HCD-MS/MS measurements (1 microscan) in the Orbitrap analyzer. Target ion quantity for FT full MS was 1×10^6^ and for MS/MS was 5×10^5^. Dynamic exclusion was enabled with a repeat count of 2 and exclusion duration of 5,000 secondes. The lock mass was enabled for accurate mass measurements. Polydimethylcyclosiloxane (m/z, 445.1200025, (Si(CH3)2O))6) ions were used for internal recalibration of the mass spectra.

For automated data acquired by µLC-MS/MS, raw files were automatically processed inside ProSightHT of the ProSight PC software v 3.0 SP1 (Thermo Fisher, San Jose). All data files (*.raw) were processed to group MS/MS data from different precursors of the same protein into one experiment for simultaneous analysis using the cRAWler application. Molecular weights of precursor and product ions were determined using the THRASH algorithm. All fragmentation data were filtered using the following parameters: signal/noise= 3/1, minimum fragment intensity at 100, retaining only the top 5 most intense neutral fragment masses within a 100 Da window below 2,000 Da., Automated searches were performed on PUF files using the “Biomarker and Absolute mass” search options against the selected database. For the biomarker searches, an iterative search tree was designed to begin with high mass accuracy (50 and 10 ppm at the intact and fragment ion level, respectively) for monoisotopic precursors. If a top result was matched with a p-score of ≤1 × 10^−6^ the search engine accepted this result as valid. A second search was performed for invalid results using larger intact mass tolerances (average precursors with 3 Da mass tolerances). For all searches, all post-translation modifications (PTM) were considered. First hits were automatically considered positively identified with a minimum of 5 matching fragment ions with E-value ≤1 × 10^−6^ for monoisotopic precursors and E-value ≤1 × 10^−8^ for average precursors. Then, all the *.puf files were additionally searched in absolute mass mode against the dataset with 1000 Da average precursor window and 10 ppm fragment tolerance.

1. µLC-HR-MS/MS with pre-fractionations

Protein extraction from about 0.8 g of GC (pool of aspirated cells from different follicles) was performed in TU buffer containing protease inhibitor cocktail as previously described (paragraph b). In order to reduce sample complexity of cellular extract, the peptides/proteins were subjected to different fractionations through reversed phase (RP) and gel filtration (GF) chromatographic separations on an UltiMate 3000 RSLC system controlled by Chromeleon version 6.80 SR13 software (Thermo Electron, Courtaboeuf, France). Additionally, one RP condition was combined to an ultracentrifugation process to enriched small proteoforms. For each condition, one mg of the peptides/proteins were injected and separated. All UV peaks (detection at 214 nm) were manually collected.

First separation method (RP1) was based on the biomolecules separation by hydrophobicity with RP HPLC using an XBridge BEH C18 column (250 × 4.6 mm i.d., particule size 5 μm; Waters, Guyancourt, France). The column temperature was maintained at 30°C. Mobile phases for the chromatographic separation consisted of (A) 0.1% (v/v) trifluoroacetic acid (TFA) in water, and (B) 0.1% (v/v) TFA in acetonitrile. The gradient elution was carried out at a flow rate of 1 mL/min with 10% B for 5 min, 60% B at 45 min, 95% B at 47 min, constant 95% B for 2 min and then back to 10 % B at 51 min. After vacuum-drying using speedVac 1010 (Thermofisher), all fractions were desalted and enriched a second time using ZT-C4-SPE (Millipore, Saint-Quentin-en-Yvelines, France) with 1% (v/v) formic acid (FA) and eluted with 50% (v/v) acetonitrile/1% (v/v) FA.

Second RP separation (RP2) was performed using a gradient elution consisting of the following concentrations of buffer B: 2% for 5 min, 60 % at 43 min, 90% at 45 min (hold for 2 min) and 2% at 49 min. Buffer A consisted of water with 0.1% TFA and buffer B consisted of 95% acetonitrile with 0.1% TFA.

Third separation (RP3) consisted to combine centrifugal ultracentrifugation using Amicon® Ultra 0.5 mL centrifugal filters with a cut-off of 50 kDa (Millipore, Saint-Quentin-en-Yvelines, France) to a RP separation. All the centrifugation steps were performed at a centrifugal force of 14,000 g for 15 min at 20°C. First, the centrifugal filter was rinsed with 0.1N NaOH followed by two rinses with water. Sample (2 mg) was diluted up to 500µL 10% (v/v) acetonitrile/5% (v/v) FA and loaded into the 50K filter device. After centrifugation, the filtrate was collected and 400 µL 10% acetonitrile/5% FA were added to the filter device. This resuspension and centrifugation process was repeated nine more times. Each time, filtrate was collected. At the end, all filtrates were pooled together and dried by vacuum-drying. The sample was dissolved in buffer A (0.1% TFA in water) and loaded onto the C18 RP-HPLC column for a RP separation under the same conditions as RP2.

Fourth separation consisted to separate peptidoforms and proteoforms by molecular weight using a Superdex 75 10/300 GL gel filtration (GF) column (GE Healthcare Europe GmbH, Velizy-Villacoublay, France) in 100 mM ammonium bicarbonate buffer. After vacuum-drying, all fractions were desalted and enriched a second time using ZipTip C4 SPE (Millipore, Saint-Quentin-en-Yvelines, France) with 1% (v/v) formic acid (FA) and eluted with 50% (v/v) acetonitrile/1% (v/v) FA.

In total, 43, 54, 46 and 40 fractions were collected from the RP1, RP2, RP3 and GF liquid chromatography processes, respectively. Samples were immediately vacuum-dried and kept at -20ºC until analyses. µLC-MS/MS analyses and TD identifications using ProSight PC software 3.0 were performed as described in the previous paragraph b, except the use of longer analytic column (Monolithic PS-DVB PepSwift, 200 µm inner diameter x 25 cm long).
